# Apolipoprotein E lipoprotein particles inhibit amyloid-β uptake through cell surface heparan sulphate proteoglycan

**DOI:** 10.1186/s13024-016-0099-y

**Published:** 2016-05-05

**Authors:** Yuan Fu, Jing Zhao, Yuka Atagi, Henrietta M. Nielsen, Chia-Chen Liu, Honghua Zheng, Mitsuru Shinohara, Takahisa Kanekiyo, Guojun Bu

**Affiliations:** Department of Neuroscience, Mayo Clinic, Jacksonville, FL USA; Fujian Provincial Key Laboratory of Neurodegenerative Disease and Aging Research, Institute of Neuroscience, College of Medicine, Xiamen University, Xiamen, Fujian China; Department of Neurology, The Fourth Affiliated Hospital, Harbin Medical University, Harbin, Heilongjiang China

**Keywords:** Aβ, apoE, HSPG, Alzheimer’s disease, Cellular uptake

## Abstract

**Background:**

The accumulation, aggregation and deposition of amyloid-β (Aβ) peptides in the brain are central to the pathogenesis of Alzheimer’s disease (AD). Alzheimer’s disease risk increases significantly in individuals carrying one or two copies of *APOE* ε4 allele compared to individuals with an ε3/ε3 genotype. Growing evidence has demonstrated that apolipoprotein E (apoE) strongly influences AD pathogenesis by controlling Aβ aggregation and metabolism. Heparan sulphate proteoglycans (HSPGs) are abundant cell surface molecules that bind to both apoE and Aβ. HSPGs have been associated with Aβ aggregation and deposition. Although several lines of research have shown that apoE influences Aβ clearance in the brain, it is not clear how apoE influences HSPG-mediated cellular uptake of Aβ.

**Results:**

In this study, we show that apoE lipoprotein particles from conditioned media of immortalized astrocytes isolated from human *APOE*-targeted replacement (TR) mice significantly suppress cellular Aβ42 and Aβ40 uptake through cell surface HSPG. ApoE3 and apoE4 particles have similar binding affinity to heparin, while apoE4 particles are likely hypolipidated compared to apoE particles. We also found that the apoE particles antagonize Aβ binding to cell surface, and inhibited Aβ uptake in a concentration-dependent manner in Chinese hamster ovary (CHO) cells. While the effect was not apoE isoform-dependent, the suppressive effect of apoE particles on Aβ uptake was not observed in HSPG-deficient CHO cells. We further demonstrated that apoE particles reduced the internalization of Aβ in mouse primary neurons, an effect that is eliminated by the presence of heparin.

**Conclusions:**

Taken together, our findings indicate that apoE particles irrespective of isoform inhibit HSPG-dependent cellular Aβ uptake. Modulating the ability of apoE particles to affect Aβ cellular uptake may hold promises for developing new strategies for AD therapy.

**Electronic supplementary material:**

The online version of this article (doi:10.1186/s13024-016-0099-y) contains supplementary material, which is available to authorized users.

## Background

The accumulation and deposition of amyloid-β (Aβ) peptides composed of 40 or 42 amino acids in the brain are thought to be central events in the pathogenesis of Alzheimer’s disease (AD) [1–4], the most common form of neurodegenerative dementia in the elderly [5]. The interaction of soluble Aβ forms, in particular oligomers, with cell surface receptors initiates a toxic cascade, leading to subsequent neurodegeneration and cognitive dysfunction [6]. On the other hand, neurons can also internalize and efficiently degrade toxic species of Aβ and eliminate these from the brain [7]. Thus, it is critical to understand how Aβ interacts with cell surface receptors and how these events impact Aβ aggregation, toxicity and/or metabolism.

The ε4 allele of the *APOE* gene encoding the apolipoprotein E (apoE) protein strongly influences AD risk and age of onset [8, 9]; risk increases significantly in individuals carrying one copy (ε2/ε4, OR 2.6; ε3/ε4, OR 3.2) or two copies (ε4/ε4, OR 14.9) of the ε4 allele compared to individuals with an ε3/ε3 genotype [10, 11]. Importantly, the occurrence of cortical Aβ deposition is increased already at early middle-age in asymptomatic *APOE* ε4 carriers [12, 13] and an age-dependent increase in Aβ deposition in the brains has been proposed as a pathobiological phenotype of *APOE* ε4 [14]. While apoE plays a critical function in lipid transport in the brain [15], apoE also directly or indirectly influences Aβ aggregation, cellular uptake, and metabolism [16, 17]. For example, apoE was shown to protect primary human pericytes and astrocytes from toxicity induced by Aβ40 harboring the Dutch mutation [18]. Cellular uptake of oligomeric versus fibrillar forms of Aβ42 is also altered by apoE in cultures of primary human astrocytes and microglia [19, 20]. Whether an apoE-mediated reduction in Aβ-uptake is beneficial or harmful is not clear; however, co-administration of apoE and Aβ42 increased the gene expression of the Aβ-degrading enzyme neprilysin in primary astrocytes isolated from post-mortem brain tissue from non-demented subjects [21]. Despite these pieces of evidence, whether interaction between apoE and Aβ represents a major pathway under physiological or pathophysiological conditions is not fully understood. Previous studies have suggested that apoE forms complexes with Aβ [6, 9] via both the receptor-binding region in the N-terminal domain and the lipid-binding region in the C-terminal domain [9, 22]. Interestingly, these Aβ-binding regions within apoE overlap with the heparin-binding regions [23, 24]. Of note, epitope mapping reveals that residues 13–17 in Aβ are common sites that interact with both apoE and heparin [22, 25]. However, a recent report showed that only a small portion of Aβ interacts with apoE lipoprotein particles in solution under physiological conditions [26]. The same study also reported the ability of apoE lipoprotein particles to compete with Aβ for cellular uptake via the low-density lipoprotein receptor-related protein 1 (LRP1) in astrocytes. Importantly, increasing the lipidation of apoE4 was recently suggested to alleviate cognitive impairment and Aβ42 accumulation in *APOE4*-targeted replacement (TR) mice suggesting that lipidation of apoE4 is key to its pathophysiological properties [27].

In the brain, the major apoE receptors are the low-density lipoprotein receptor (LDLR) [28] and LDLR-related protein 1 (LRP1) [29, 30]. In addition, heparan sulphate proteoglycan (HSPG) on the cell surface also mediates cellular uptake of apoE lipoprotein particles, either independently or in coordination with LRP1 [31]. Heparan sulphate proteoglycan is abundantly expressed on the cell surface and functions as a receptor that interacts with a variety of ligands through electrostatic interactions [32]. In conjunction with LRP1, HSPG plays an important role in neuronal cellular uptake of Aβ, which also possesses a heparin-binding region [25, 33]. HSPG controls the entry of a variety of molecules on the cell surface, including tau, α-synuclein and soluble APP [34, 35].

In this study, we aimed to elucidate whether the previously reported apoE-mediated decrease in cellular Aβ-binding and uptake is orchestrated through competition between apoE and Aβ for HSPG binding. We further aimed to clarify if such an interaction is apoE isoform-dependent. For these purposes we used immortalized astrocyte-produced human apoE3 and apoE4 lipoprotein particles, Chinese hamster ovary (CHO) cells, with or without HSPG, and primary cultured neurons. We show that apoE3 and apoE4 particles in a similar way irrespective of lipidation status compete with cellular Aβ binding and uptake in a manner that depends on cell surface HSPG.

## Results

### Astrocyte-secreted apoE3 particles carry more lipids than apoE4 particles

**Fig. 1:**
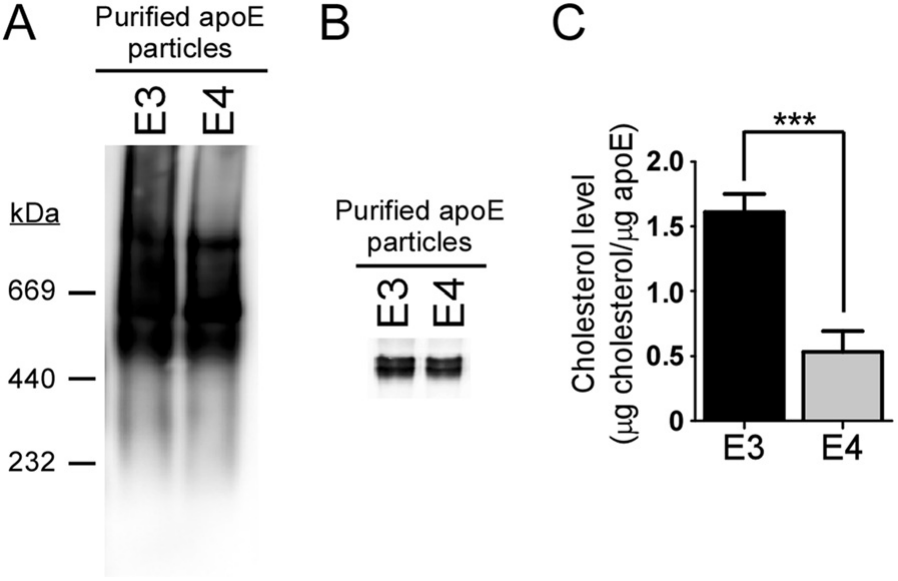
Characterization of astrocyte-secreted apoE particles. Immunoaffinity purified apoE3 (500 ng) and apoE4 particles (500 ng) were analyzed by non-denaturing gradient gel electrophoresis (4–20 %) (**a**) and SDS-PAGE (**b**) followed by Western blot for apoE. Numbers on the left are molecular size markers expressed in kDa. **c** Total cholesterol concentrations of apoE3 and apoE4 particles were measured using the Amplex Red cholesterol assay kit and normalized against apoE concentrations. Data represent mean ± S.D. (*n* = 3). ***, *p* < 0.001

Western blot of apoE3 and apoE4 after separation by nondenaturing gel revealed high molecular weight particles (>232 kDa) in both the apoE3 and apoE4 preparations with a slightly higher presence of higher molecular weight particles (>669 kDa) in the apoE3 preparation (Fig. 1a). Upon separation of apoE3 and apoE4 under reducing conditions, both apoE3 and apoE4 were detected at the expected molecular size of monomeric apoE (~37 kDa) (Fig. 1b). Employing a total cholesterol quantification assay, the total cholesterol levels in the apoE3 particle preparation was found to be 1.61 ± 0.12 μg cholesterol/μg apoE, whereas the apoE4 particle preparation contained 0.53 ± 0.13 μg cholesterol/μg apoE (Fig. 1c). Similar results were obtained using apoE3 and apoE4 particles secreted by primary astrocytes from apoE-TR mice (Additional file 1: Figure S1). Together, these results indicate that apoE produced by astrocytes exists as lipoprotein particles with apoE3 particles carrying more cholesterol compared to apoE4 particles.

### ApoE binds to heparin and requires HSPG for cell internalization

**Fig. 2:**
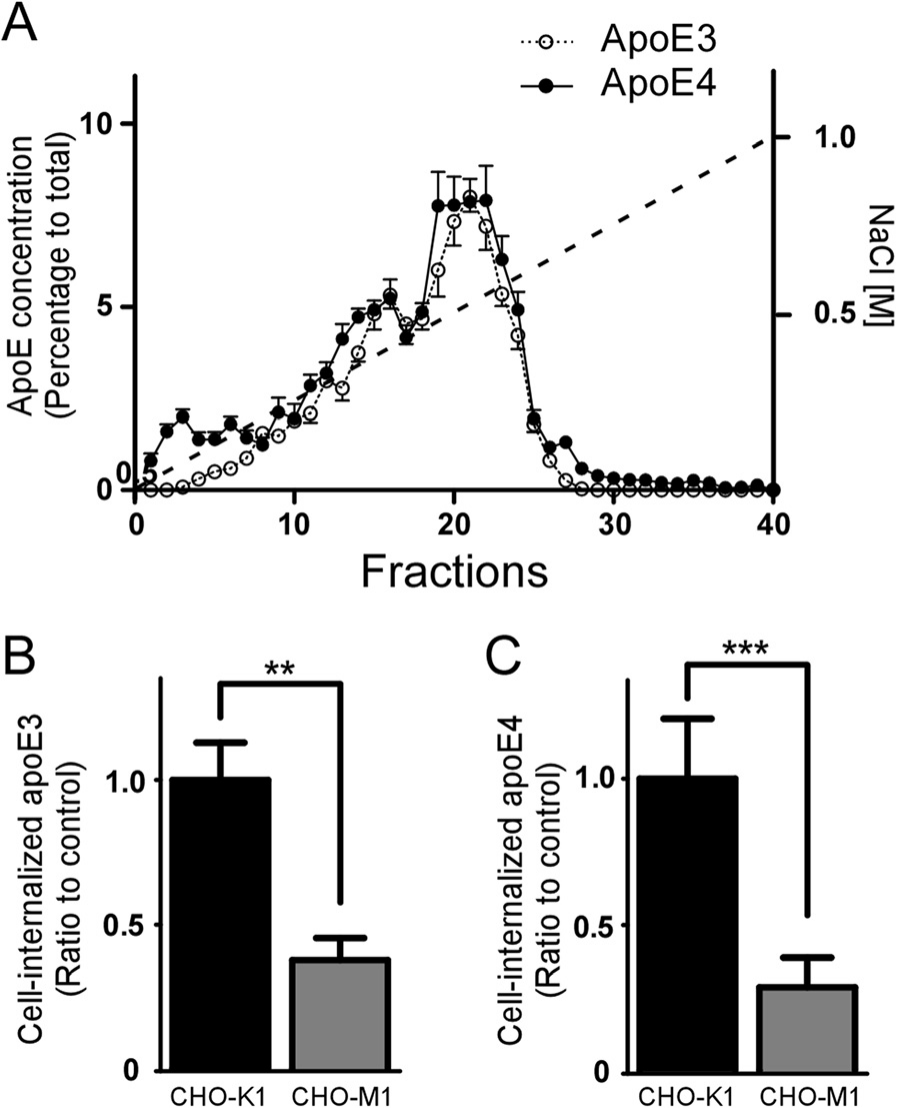
ApoE particles bind to heparin and cell surface HSPG. ApoE3 particles (3.4 μg) and apoE4 particles (3.4 μg) were applied to HiTrap Heparin HP columns attached to a FPLC and eluted with a linear salt gradient (0–1.0 M NaCl). Concentrations of apoE in each fraction were determined by ELISA (**a**). An average of *n* = 3 experiments are plotted in the graph. CHO-K1 or CHO-M1 cells were incubated with apoE3 (200 nM) (**b**) or apoE4 (200 nM) (**c**) for 18 h at 37 °C. Internalization of apoE was analyzed by ELISA. Data represent mean ± S.D. (*n* = 3). **, *p* < 0.01; ***, *p* < 0.001

To determine whether immortalized astrocyte-produced apoE particles bind to heparin, we injected purified apoE3 and apoE4 particles into a Hitrap heparin HP column attached to FPLC and eluted apoE with a linear gradient of increasing concentrations of NaCl. The concentration of apoE in each fraction was quantified by ELISA. ApoE3 particles and apoE4 particles bound to heparin column and were effectively eluted with NaCl. The peaks of both apoE isoforms were detected after elution with a supraphysiological NaCl concentration of 0.45 M, which showed similar patterns with no obvious difference in the elution profiles between apoE3 and apoE4 particles (Fig. 2a). These results suggest that apoE particles, independent of isoform, strongly interact with heparin at NaCl concentrations <0.45 M and thus apoE likely binds to cell surface HSPG under physiological conditions. Furthermore, to determine the kinetics of apoE-heparin binding, their binding affinity was analyzed by dot blot. The dissociation constant (Kd) value between apoE3 particles and heparin was calculated as ~201 nM (Additional file 2: Figure S2).

To further confirm the role of HSPG in apoE cellular uptake, we used wild-type CHO-K1 cells and CHO-M1 cells lacking N-acetylglucosaminyltransferase/glucuronyltransferase, which is required for the biosynthesis of heparan sulphate [36]. When CHO-K1 or CHO-M1 cells were incubated with apoE3 or apoE4 particles (200 nM) for 18 h, the amounts of cell-associated apoE were 62 % (Fig. 2b) and 71 % (Fig. 2c) lower in CHO-M1 cells than those of CHO-K1 cells. These results indicate that HSPG is a major receptor facilitating the cellular uptake of astrocyte-secreted apoE particles in CHO cells.

### ApoE particles compete with Aβ for internalization through HSPG

**Fig. 3:**
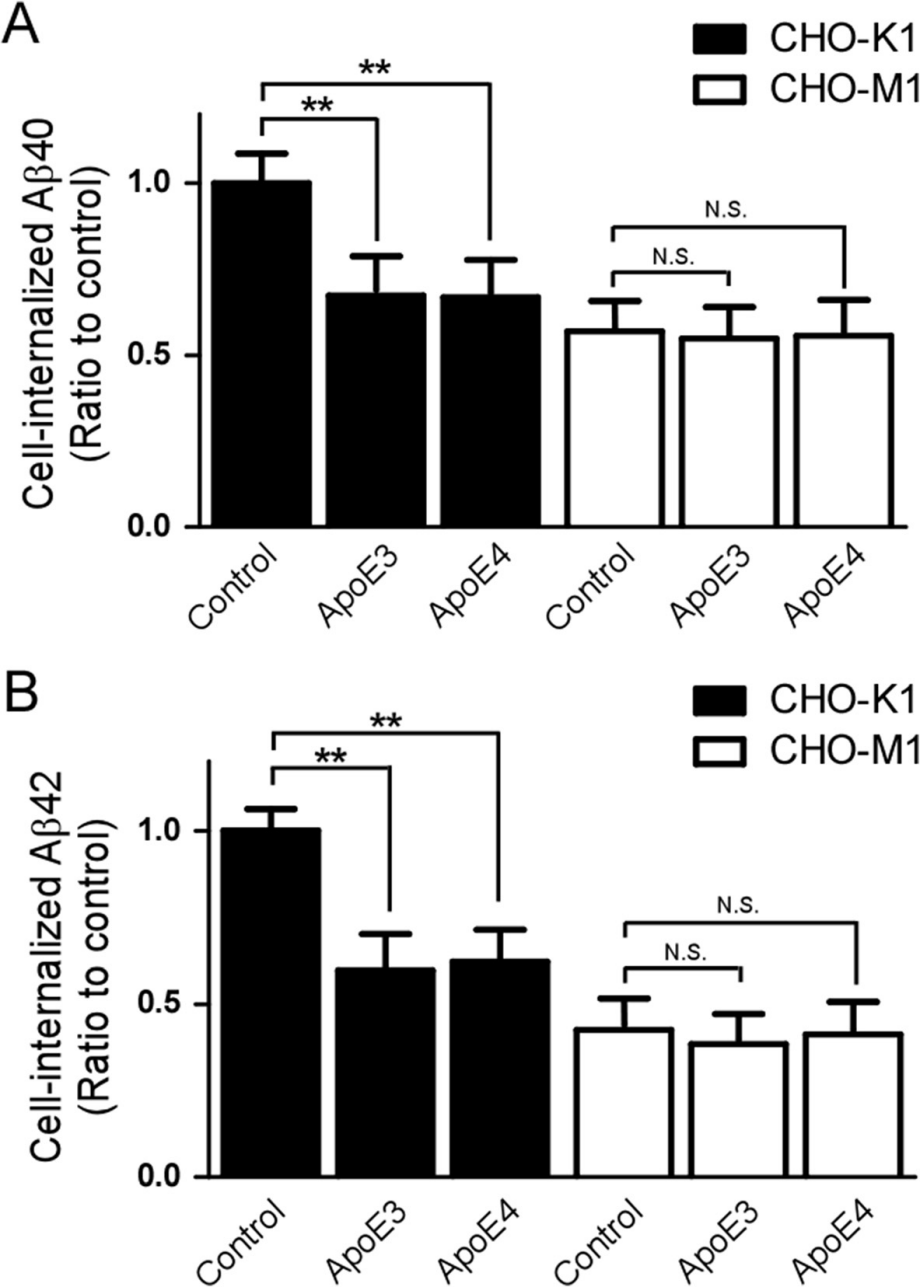
ApoE particles inhibit Aβ cellular uptake through HSPG in CHO cells. CHO-K1 (wild-type) cells or CHO-M1 (HS-deficient) cells were incubated with 50 nM Aβ40 (**a**) or 50 nM Aβ42 (**b**) in the presence or absence of apoE3 particle (200 nM) or apoE4 particle (200 nM) for 18 h at 37 °C. The amount of internalized Aβ was quantified by ELISA. Data represent mean ± S.D. (*n* = 3). **, *p* < 0.01

**Fig. 4:**
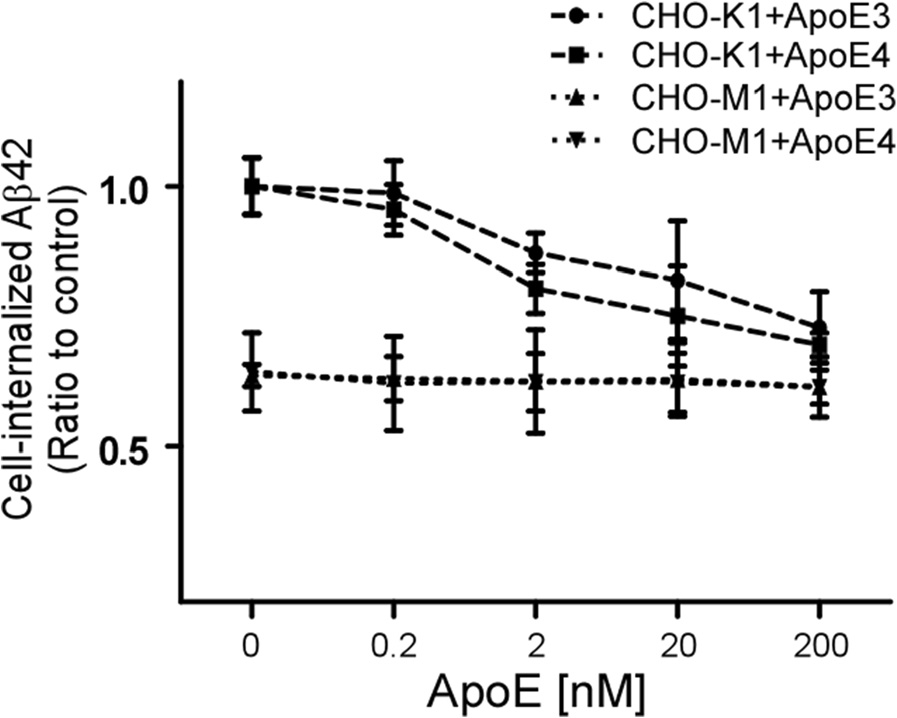
ApoE particles inhibit HSPG-mediated cellular uptake of Aβ in a concentration-dependent manner. CHO-K1 cells or CHO-M1 cells were incubated with Aβ42 (50 nM), together with various concentrations of apoE3 particles (0.2 nM to 200 nM) or apoE4 particles (0.2 nM to 200 nM) for 18 h at 37 °C. The amount of internalized Aβ was quantified by ELISA. Data represent mean ± S.D. (*n* = 3)

**Fig. 5:**
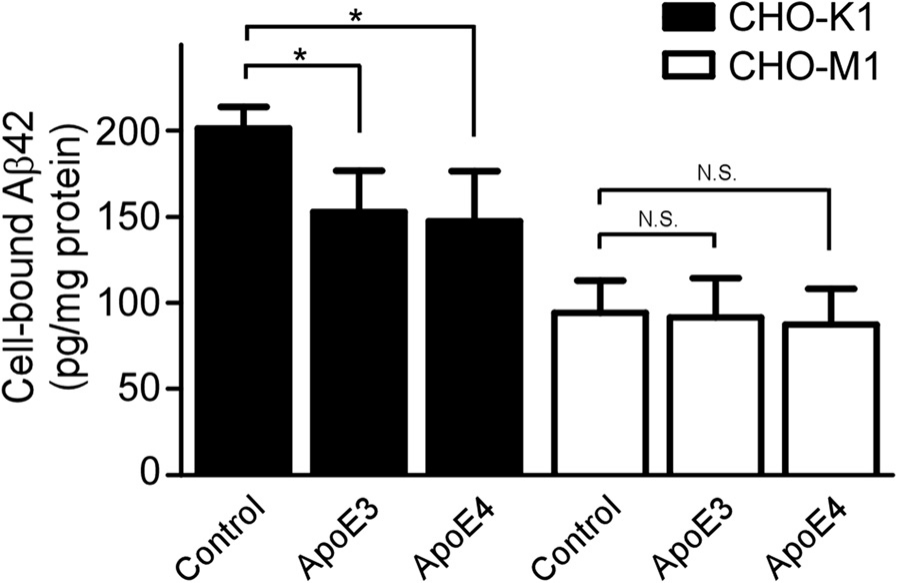
ApoE particles suppress Aβ binding to HSPG on the cell surface. CHO-K1 or CHO-M1 cells were incubated with Aβ42 (50 nM) with or without apoE3 particles (200 nM) or apoE4 particles (200 nM) for 3 h at 4 °C. Cell-bound Aβ42 was quantified by ELISA. Data represent mean ± S.D. (*n* = 3). *, *p* < 0.05

To investigate effects of apoE on cellular Aβ uptake, CHO cells were incubated with 50 nM of Aβ40 or Aβ42 in the presence or absence of apoE particles for 18 h at 37 °C. The amount of cell-associated Aβ was assessed by ELISA. We found that the amounts of Aβ40 (Fig. 3a) and Aβ42 (Fig. 3b) were significantly reduced by both apoE3 and apoE4 particles (200 nM) in CHO-K1 cells. The internalization of both Aβ42 and Aβ40 in CHO-M1 cells was significantly lower than in CHO-K1 cells, consistent with a role of cell surface HSPG in cellular Aβ uptake [33]. More importantly, the effects of apoE particles on cellular Aβ uptake were completely abolished in CHO-M1 cells. To further interrogate the effects of apoE particles on cellular Aβ uptake, we performed a competition assay using fixed concentration of Aβ42 (50 nM) and increasing concentrations of apoE particles (0.2 nM to 200 nM) in CHO cells. Both apoE isoforms significantly suppressed Aβ42 internalization in CHO-K1 cells in a concentration-dependent manner (Fig. 4). These results indicate that apoE particles compete with Aβ for cellular uptake in a manner that depends on cell surface HSPG. We next investigated whether apoE affects Aβ binding to cell surface HSPG. CHO-K1 cells and CHO-M1 cells were incubated with Aβ42 (50 nM) in the presence or absence of astrocyte-secreted apoE (200 nM) at 4 °C for 3 h. When the amount of cell-associated Aβ was analyzed by ELISA, approximately 1 % of the added Aβ was bound to the cells. We found that both apoE3 and apoE4 particles in a similar manner inhibited Aβ binding to the cell surface in CHO-K1 cells. However, these effects were absent in CHO-M1 cells (Fig. 5), indicating that apoE particles reduce Aβ binding to cell surface HSPG.

### ApoE particles inhibit HSPG-mediated cell-association of Aβ in primary neurons

**Fig. 6:**
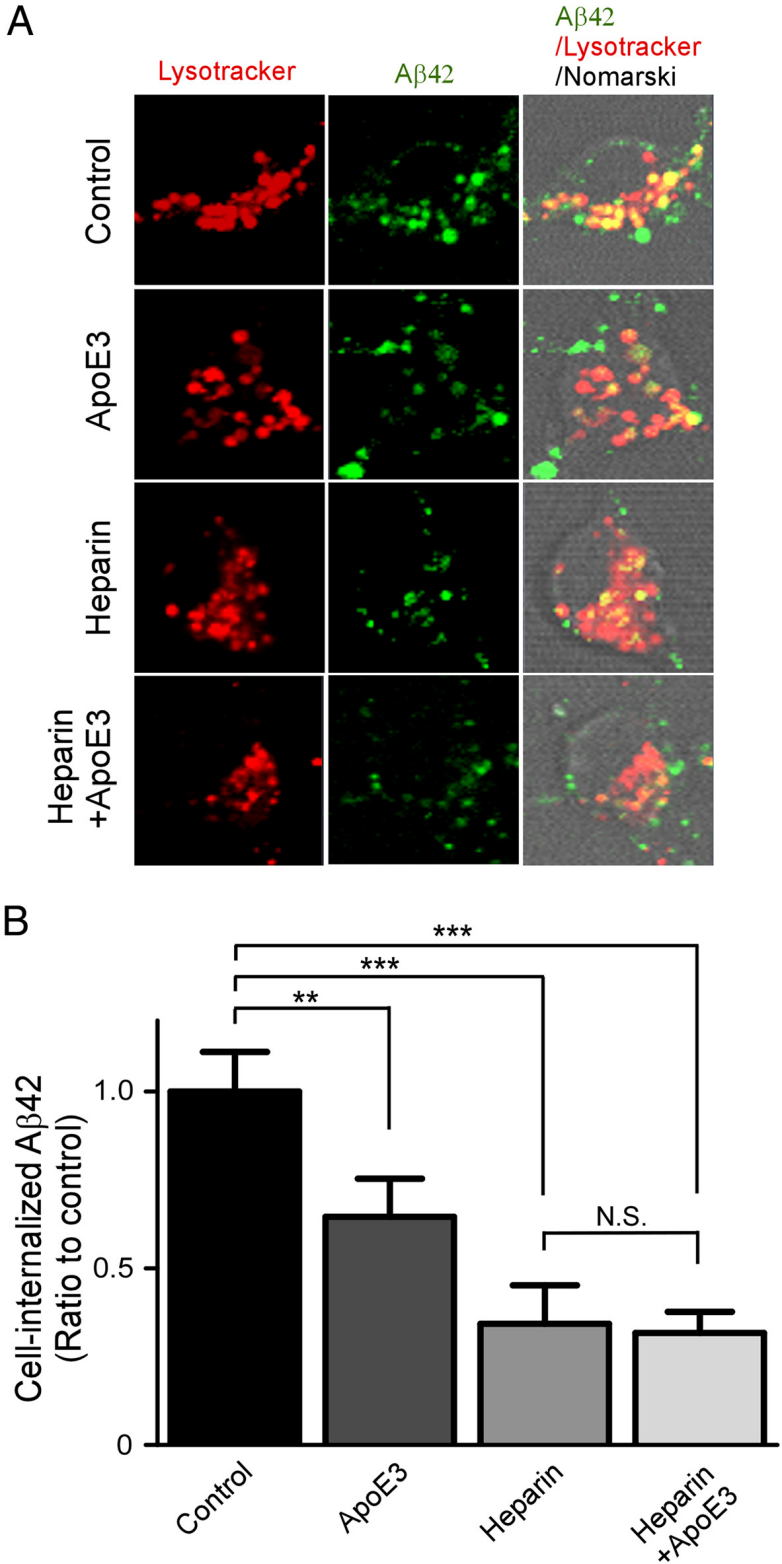
ApoE particles decrease HSPG-mediated cellular uptake of Aβ in primary neurons. **a** Mouse primary cortical neurons were incubated with FAM-Aβ42 (500 nM) with or without apoE3 particles (200 nM) in the presence or absence of heparin (15 U/ml) for 6 h at 37 °C and analyzed by confocal microscopy. Left, middle, and right columns indicate Lysotracker, FAM-Aβ42, and merged images, respectively. **b** Internalization of Aβ was quantified by FACS after incubation with FAM–Aβ42 (500 nM) for 6 h. Data represent mean ± S.D. (*n* = 3). *N.S.*, not significant; **, *p* < 0.01; ***, *p* < 0.001

To determine whether the observed effects of apoE particles on Aβ uptake are also relevant in neurons, we analyzed the effects of apoE3 particles on Aβ uptake in the presence or absence of heparin in mouse primary cortical neurons, as there is no detectable difference between apoE3 and apoE4 particles on Aβ uptake in CHO-cells. Neurons were incubated with Aβ42 in the presence or absence of apoE3 particles with or without heparin to antagonize HSPG binding for 6 h at 37 °C and analyzed by confocal microscopy (Fig. 6a). When neuronal cell bodies were observed by confocal microscopy, internalized Aβ was mainly co-localized with a lysosomal marker, LysoTracker. In the presence of apoE3 particles or heparin, Aβ42 internalization was reduced compared to the controls (Fig. 5). The effect of apoE3 particles on Aβ uptake was not observed when heparin was co-incubated (Fig. 5). Consistent with the results from confocal microscopy, FACS revealed that apoE3 particles and heparin significantly suppressed cell-associated Aβ42 levels. More importantly, the effects of apoE3 particles on Aβ42 uptake were eliminated by heparin (Fig. 6b). Together, these results indicate that apoE particles inhibit neuronal Aβ42 uptake though their binding to cell surface HSPG.

## Discussion

Interaction of Aβ with the neuronal cell surface significantly affects synaptic functions and may induce neuronal toxicity in AD pathogenesis [37, 38]. The binding of Aβ to synapses alters the distribution and/or activities of signaling receptors including N-methyl D-aspartate (NMDA) receptors [39] and metabotropic glutamate receptors (mGluR5) [40], leading to synapse deterioration and impairment of learning and memory. Oligomeric Aβ also binds to prion protein (PrPc), resulting in significant synaptic dysfunction [41]. Cell-surface HPSG may be heavily involved in these processes as HSPG mediates binding and uptake of aggregated Aβ and monomeric Aβ on the cell surface [33, 42, 43]. HSPGs are composed of a core protein, several heparan sulphate [44] glycosaminoglycan [44, 45] chains, and uronic acid [46]. Heparan sulphate chains, which have a high negative charge, presumably interact with the binding sites in the protein with positive charges [46].

Receptor-mediated cellular uptake and subsequent lysosomal degradation is an important pathway for Aβ clearance [47]. For example, LRP1 regulates cellular Aβ uptake in neurons [48] and vascular smooth muscle cells [49], and the disturbances of this pathway exacerbates amyloid pathology in amyloid mouse models. ApoE is also likely to interact directly with LRP1 and competes with Aβ in the cellular clearance process at their physiological concentrations [26]. These observations support previous findings that apoE may have a suppressive effect from the perspective of cellular Aβ elimination by inhibiting cellular Aβ clearance pathways in both rodent astrocytes [26] and primary human astrocytes and microglia [19, 20, 26]. Interestingly, the clearance of soluble Aβ in brain interstitial fluid is significantly accelerated in *Apoe*-KO mice [16] and decreased apoE expression under haploinsufficiency also results in less Aβ deposition in amyloid mouse models expressing human apoE isoforms [50, 51]. Further, immunotherapy for apoE also reduces Aβ accumulation in rodent models [52, 53].

Although binding of Aβ to cell surface HSPG can lead to its subsequent trafficking to lysosomes for degradation, there is also strong evidence that such interaction can be harmful by promoting Aβ aggregation and oligomer formation [54, 55]. Several studies have shown that soluble Aβ oligomers injure synapses resulting in impairment of cognitive function through direct binding, and the levels of Aβ in the brain of AD patients are positively associated with synaptic protein and cognitive decline [44, 56, 57]. In these cases, inhibition of Aβ binding to cell surface HSPG by apoE particles can be beneficial by reducing the formation of harmful Aβ oligomers. Mounting evidence has shown that the *APOE ε4* allele dramatically increases the risk for AD, where apoE4 contributes to AD pathogenesis by both loss-of-function in neuroprotection and gain-of-function in neurotoxicity compared to apoE3 [47, 58]. Although isoform-dependent effects were not detected in the current study, apoE particles blocked the internalization of Aβ in a concentration-dependent manner. As apoE indeed could act as an inducer of Aβ fibril formation with apoE4 exhibiting the strongest fibril catalytic activity [59], apoE isoforms may differently modulate Aβ aggregation status which influences Aβ proteolytic degradation, rather than affecting cellular Aβ uptake. In addition, we found that our purified apoE4 particles contained less cholesterol than apoE3 particles, which is consistent with earlier studies proposing apoE4 as a less efficient lipid-carrier [27, 60]. Thus, the less lipidation status of apoE4 may also be involved in the exacerbated Aβ plaque accumulation through apoE4-induced Aβ aggregation.

Furthermore, we found that apoE particles compete with Aβ for HSPG binding and subsequent cellular uptake in CHO cells and neurons. By applying apoE3 and apoE4 to a heparin HiTrap column, we found that both apoE3 and apoE4 bound tightly to heparin at physiological NaCl concentrations (<0.45 M). Consistent with our results, surface plasmon resonance failed to detect significant differences between apoE isoforms for their bindings to heparin [61]. Another study has also demonstrated that there are no significant differences in rate and equilibrium constants of binding among the lipidated apoE isoforms to heparin [24]. Since apoE has heparin binding sites in both the N-terminal and C-terminal domains with binding motifs identical between apoE3 and apoE4 [23, 24], it is not surprising that these apoE isoforms have similar binding affinities to heparin. Using dot blot, we also investigated whether the astrocyte-secreted apoE3-heparin affinity constant was similar to apoE-heparin binding constants previously reported for recombinant and plasma-derived apoE [24, 61, 62]. We estimated the Kd to be 201 nM which resembles the apoE3-heparin constant reported by Libeu and colleagues who found a Kd of 320 nM as determined by surface plasmon resonance [62], however recombinant apoE was reported to bind heparin with higher affinity than that we determined in the current study [61].

Previously Aβ was proposed to bind and form stable complexes with apoE [9, 63, 64]; however, under physiological conditions only a small portion of Aβ, estimated at less than 5 % of total Aβ, was found to interact with apoE particles [26]. It was proposed that despite minimal interaction between apoE and Aβ, these two players in solution compete for cellular uptake by astrocytes. Consistent with that report, we also found limited molecular interaction between Aβ (50 nM) and apoE (200 nM) as assessed using SEC under which Aβ and apoE eluted in separate fractions when quantified by ELISA (Additional file 3: Figure S3). These seemingly conflicting results in regard to apoE-Aβ binding may be due to various studies employing different conditions, such as the concentrations of apoE and Aβ, their aggregation state and apoE lipidation state. It has been shown that CSF concentrations of apoE range between 2 and 7 μg/ml (~200 nM) [65, 66], whereas the interstitial fluid concentrations of Aβ were found to be up to ~2000 pg/ml (~0.5 nM) [67]. Although the focal concentration of apoE and Aβ remain unclear in particular in the synaptic region, our results suggest that apoE does not or only minimally interact with Aβ under our experimental conditions.

In humans, higher levels of apoE tissue concentrations were previously shown to correlate with lower Aβ burden in human brain homogenates [68] and increased CSF apoE levels were associated with higher CSF Aβ42 levels indicative of lower Aβ plaque burden [66]. Indeed, increasing apoE by retinoid X receptor (RXR) and liver X receptor (LXR) agonist has been shown to decrease Aβ deposition in amyloid mouse models [69, 70]. We also recently reported that retinoic acid (RA) isomers including all-trans-RA, 9-cis-RA, and 13-cis-RA can increase the astrocyte-secreted apoE level through the RXR/retinoic acid receptor pathway and simultaneously decreased the amount of cell-associated Aβ in immortalized astrocytes [71]. Thus, the regulation of brain apoE levels through these receptors likely contributes to Aβ metabolism in AD pathogenesis.

## Conclusions

Overall, our findings support previous reports showing an inhibitory effect of apoE on cellular Aβ uptake and further extend those results by demonstrating that both apoE3 and apoE4 particles similarly reduce cellular Aβ uptake through interactions with cell surface HSPG in CHO cells and neurons. In addition, apoE lipidation status may not contribute to its inhibitory effect on cellular Aβ binding and uptake. Together these findings propose potent effects of apoE on Aβ clearance possibly by inhibiting cellular uptake which may promote the keeping of Aβ in the extracellular space readily available for proteolytic degradation. The interaction between apoE and HSPG may be critical to the development of AD pathology by modulating the extra- versus intracellular pools of Aβ with down-stream effects on several AD-related pathways. Our findings propose that modulating the interaction between apoE, Aβ and HSPG may be an attractive target to be explored for AD therapy.

## Methods

### Reagents

5(6)-Carboxyfluorescein (FAM)-Aβ42, synthetic Aβ40, and synthetic Aβ42 were purchased from AnaSpec (Fremont, CA). LysoTracker Red was purchased from Invitrogen. Heparin was purchased from Baxter Healthcare Corp. (Round Lake, IL).

### Preparation of apoE particles

Immortalized apoE astrocyte cell lines producing human apoE3 or apoE4, kind gifts from Dr. David Holtzman [72], were cultured in DMEM/F12 (Invitrogen) with 20 % fetal bovine serum (Gibco), 1 mM sodium pyruvate, 1x non-essential amino acids, 2 mM L-glutamine, and 1 % penicillin/streptomycin (Invitrogen). This cell line was generated from primary astrocytes derived from human *APOE*-TR mice, in which human *APOE* gene is knocked into the mouse *Apoe* locus. Cells were cultured at 37 °C in humidified air containing 5 % CO_2_. For purification of apoE lipoprotein particles, WUE-4, a mouse monoclonal antibody to human apoE [73], was coupled to CNBr-activated Sepharose 4B (GE Healthcare) and immunoaffinity columns were prepared as described [74]. ApoE lipoprotein particles were then purified from conditioned serum-free media of immortalized astrocytes by using apoE antibody columns as described [72].

### Western blot

Samples were separated by electrophoresis on either a nondenaturing 4–20 % polyacrylamide gradient gel or on a 10 % sodium dodecyl sulfate (SDS)-polyacrylamide gel (PAGE) as described [73], transferred to nitrocellulose membrane, and probed with a monoclonal antibody to human apoE (WUE-4) followed by an HRP-conjugated secondary antibody. The immunoreactive proteins were detected using enhanced chemiluminescence (ECL, Thermo) with images obtained with the Fujifilm® LAS-4000 gel imager.

### Cholesterol assay

Total cholesterol contents of the apoE3 and apoE4 particles were quantified with the Amplex Red cholesterol assay kit (Invitrogen) according to the manufacturer’s protocol.

### Heparin-apoE interaction by affinity chromatography

Binding of apoE lipoprotein particles to heparin was assessed using a heparin column as described previously [75]. In brief, a 1 ml HiTrap heparin HP column (GE Healthcare) was attached to a fast protein liquid chromatography (FPLC) (GE Healthcare) and run at a flow rate of 1 ml/min. 3.4 μg of apoE3 or apoE4 were loaded, and the column was washed with 20 mM Tris–HCl, pH 7.4 for 10 column volumes. Bound apoE was eluted in the same buffer using a linear gradient of 0–1.0 M NaCl.

### Heparin-apoE interaction by dot-blot analysis

WUE-4 (mouse monoclonal anti-apoE antibody), normal mouse IgG (Invitrogen), and heparin (Sigma-Aldrich) were spotted onto a nitrocellulose membrane (Bio-Rad) using dot blot manifold apparatus (GE Healthcare). Membrane strips were then incubated with increasing concentrations of astrocyte-produced apoE3 particles overnight at 4 °C. ApoE bound to the membrane was detected with biotin-conjugated goat anti-apoE (Meridian Life Science) and Streptavidin secondary antibodies. Blots were imaged and quantified using Odyssey Infrared Imaging System (LI-COR Biosciences). For determination of binding affinity, the integrated infrared signal (K Count) of each dot was analyzed using Prism (Graphpad).

### Cell culture

Wild-type (K1) and HSPG-deficient (M1) Chinese hamster ovary (CHO) cells were grown in Ham’s F-12 medium containing 10 % fetal bovine serum (Gibco) and 1 % penicillin/streptomycin (Invitrogen). Primary cortical neurons were isolated from 17 days old embryos of wild-type C57BL/6 mice and grown in Neurobasal medium (Invitrogen) supplemented with 0.5 mM GlutaMax (Invitrogen), and B27 (Invitrogen) [33, 76].

### Primary astrocyte cultures

Primary astrocytes were prepared from new-born pups (P1-P2) with apoE3-TR and apoE4-TR background. In brief, the brain was removed from the skull and the meninges were discarded. Subsequently, the cortices were minced and incubated with 0.05 % trypsin at 37 °C for 15 min. Enzyme-digested dissociated cells were triturated with astrocyte growth medium and centrifuged at 300 × g for 5 min. The cell pellet was resuspended in fresh medium, passed through a 70-μm nylon mesh, and centrifuged at 300 × g for 5 min. The cells were plated on poly-D-lysine–coated 75-flask in the media, which were changed every three days. Cells were grown until confluence. Medium was collected and purified using the same method described above.

### Membrane protein extraction

Membrane protein was extracted according to the protocol of Thermo Scientific Mem-PER Plus Membrane Protein Extraction Kit. Briefly, 5 × 10^6^ cells were suspended in the growth media by scraping the cells off the surface of the plate with a cell scraper. After centrifugation at 300 × g for 5 min, cell pellet was resuspended in 1.5 ml of Cell Wash Solution, and centrifuged at 300 × g for 5 min. The pellet was resuspended in 0.75 ml of Permeabilization Buffer, and incubated for 10 min at 4 °C with constant mixing. After centrifugation for 15 min at 16,000 × g, the pellet was dissolved in 0.5 ml of Solubilization Buffer and incubated at 4 °C for 30 min with constant mixing. Supernatant containing solubilized membrane and membrane-associated proteins was collected after centrifugation at 16,000 × g for 15 min at 4 °C, and used for Western Blot. Protein was loaded after normalized with the level of Na^+^-K^+^-ATPase. Level of LRP1 was analyzed in the membrane fractions isolated from CHO-K1 and CHO-M1 cells (Additional file 4: Figure S4).

### Detection of cell-associated Aβ and apoE by ELISA

Cells were incubated with Aβ42 (50 nM) or Aβ40 (50 nM) in the presence of various concentrations of apoE3 or apoE4 (0.2-200 nM) in serum free medium for 18 h at 37 °C, and harvested by incubating with trypsin for 5 min at 37 °C. After washing two times with PBS, cells were dissolved in 5 M guanidine in 50 mM Tris–HCl (pH 8.0). To quantify cellular internalization of human Aβ42, Aβ42 was captured with mAb 2.1.3 antibody followed by detection with HRP-conjugated Ab5 antibody [76]. To determine human apoE concentrations, apoE was captured using apoE monoclonal antibody WUE-4 [73], detected with biotin-conjugated goat anti-apoE antibody (Meridian Life Science) and poly-HRP-conjugated streptavidin (Fitzgerald) essentially as previously described [71]. To assess cell-bound Aβ, cells were incubated with Aβ42 (50 nM) with or without apoE3 particles (200 nM), or apoE4 particles (200 nM), for 3 h at 4 °C in PBS with 1.5 % FBS after suspension by Cell Dissociation Solution. After washing three times with PBS, cells were dissolved in 5 M guanidine in 50 mM Tris–HCl (pH 8.0) The Aβ concentration in the cell lysate was quantified by ELISA and normalized by the protein concentration of the cell lysate [33].

### Size-exclusion chromatography (SEC) to determine apoE-Aβ interaction

Immunopurified apoE3 (200 nM) was incubated in serum-free DMEM-F12 medium containing Aβ (50 nM) for 3 h. The sample was loaded on SEC by fast protein liquid chromatography (FPLC) using tandem Superose-6, 10/300 GL columns (GE Healthcare) in phosphate buffer containing 50 mM sodium phosphate (pH 7.4), 150 mM NaCl, 1 mM EDTA, and 0.02 % sodium azide [71]. To determine a potential association between apoE and Aβ, the concentrations of both apoE and Aβ in each fraction were determined by ELISA as described above.

### Confocal imaging of neuronal Aβ uptake

Mouse primary cortical neurons were cultured on eight-well chambered cover glasses (Nalge Nunc International, Rochester, NY). After incubation with FAM-Aβ42 (500 nM) at 37 °C for 6 h, together with or without apoE3 particles (200 nM) in the presence or absence of heparin (15 U/ml), fluorescence associated with Aβ was observed by confocal laser-scanning fluorescence microscopy (model LSM510 invert; Carl Zeiss, Jena, Germany). LysoTracker (50 nM; Invitrogen) was added to determine lysosome-Aβ colocalization 30 min before confocal imaging.

### Fluorescence-activated cell sorter-based Aβ internalization assays

Mouse primary cortical neurons were incubated with FAM-Aβ42 (500 nM), together with vehicle or apoE3 (200 nM) in the presence or absence of heparin at 37 °C for 6 h. Cell-surface Aβ was removed using 0.25 % trypsin/EDTA (Invitrogen), and cell-associated Aβ was analyzed for fluorescence on a BD FACSCalibur (BD Biosciences). Control cells without any exposure to fluorescence were used to assess background fluorescence [33].

### Statistical analysis

All quantified data represent an average of triplicate samples and were analyzed by either *t*-test or one-way ANOVA with a Tukey’s posttest. Error bars represent standard deviation and *p* <0.05 was considered significant.

## Additional files

Additional file 1: Figure S1. ApoE particles secreted by primary astrocytes from apoE-TR mice. (A) ApoE3 and apoE4 particles were isolated from condition medium of primary astrocytes from apoE3-TR and apoE4-TR mice using immunoaffinity column. The purified apoE3 (500 ng) and apoE4 particles (500 ng) were analyzed by non-denaturing gradient gel electrophoresis (4–20 %) and SDS-PAGE, followed by Western blot for apoE. (B) Total cholesterol concentrations of the apoE3 and apoE4 particles were measured using the Amplex Red cholesterol assay kit and normalized against apoE concentrations. Data represent mean ± S.D. (*n* = 3). **, *p* < 0.001. (TIF 213 kb) Additional file 2: Figure S2. Binding affinity of heparin-apoE3 interaction. (A) Representative dot blot of heparin and apoE3 particles. Heparin was spotted onto nitrocellulose membrane along with mouse monoclonal anti-apoE antibody, WUE4, as a positive control and normal mouse IgG as a background. Membrane strips were incubated with increasing concentrations of apoE3 particles from immortalized astrocytes. Membrane-bound apoE was then visualized by biotin-conjugate anti-apoE antibody and infrared streptavidin secondary antibody. (B) Integrated infrared signal intensities from each dot were obtained and the average intensities from three independent experiments were plotted to acquire binding affinity curve and the dissociation constant (Kd). (TIF 2432 kb) Additional file 3: Figure S3. ApoE particles are minimally associated with Aβ. Aβ was incubated without (A) or with apoE3 particles (B). Samples were applied to SEC with tandem Superose-6 columns. Concentrations of apoE and Aβ in each fraction were analyzed by ELISA. Graphs are averaged from separate experiments (*n* = 3). (TIF 4171 kb) Additional file 4: Figure S4. Similar LRP1 levels in the membrane fractions of CHO-K1 cells and CHO-M1 cells. Membrane fractions isolated from CHO-K1 and CHO-M1 cells were subjected to Western blot for LRP1 and Na-K-ATPase (A). The levels of LRP1 were normalized against those of Na^+^-K^+^-ATPase and plotted in (B). Data represent mean ± S.D. (*n* = 3). N.S., not significant. (TIF 63 kb)

## Abbreviations

Aβ: amyloid-β
AD: Alzheimer’s disease
ApoE: apolipoprotein E
HSPG: heparan sulphate proteoglycan
apoE-TR mice: apoE targeted-replacement mice
CHO: Chinese hamster ovary
LRP1: low-density lipoprotein receptor-related protein 1
LDLR: low-density lipoprotein receptor
APP: amyloid precursor protein
HDL: high density lipoprotein
NMDA: N-methyl d-aspartate
mGluR5: metabotropic glutamate receptors
PrPc: prion protein
CSF: cerebrospinal fluid
RXR: retinoid X receptor
LXR: liver X receptor
RA: retinoic acid
