# Supplementary figures and images for: Apolipoprotein E lipoprotein particles inhibit amyloid-β uptake through cell surface heparan sulphate proteoglycan
Source: Mol Neurodegener. 2016 May 5;11:37. doi: 10.1186/s13024-016-0099-y (PMC4857252; doi:10.1186/s13024-016-0099-y)

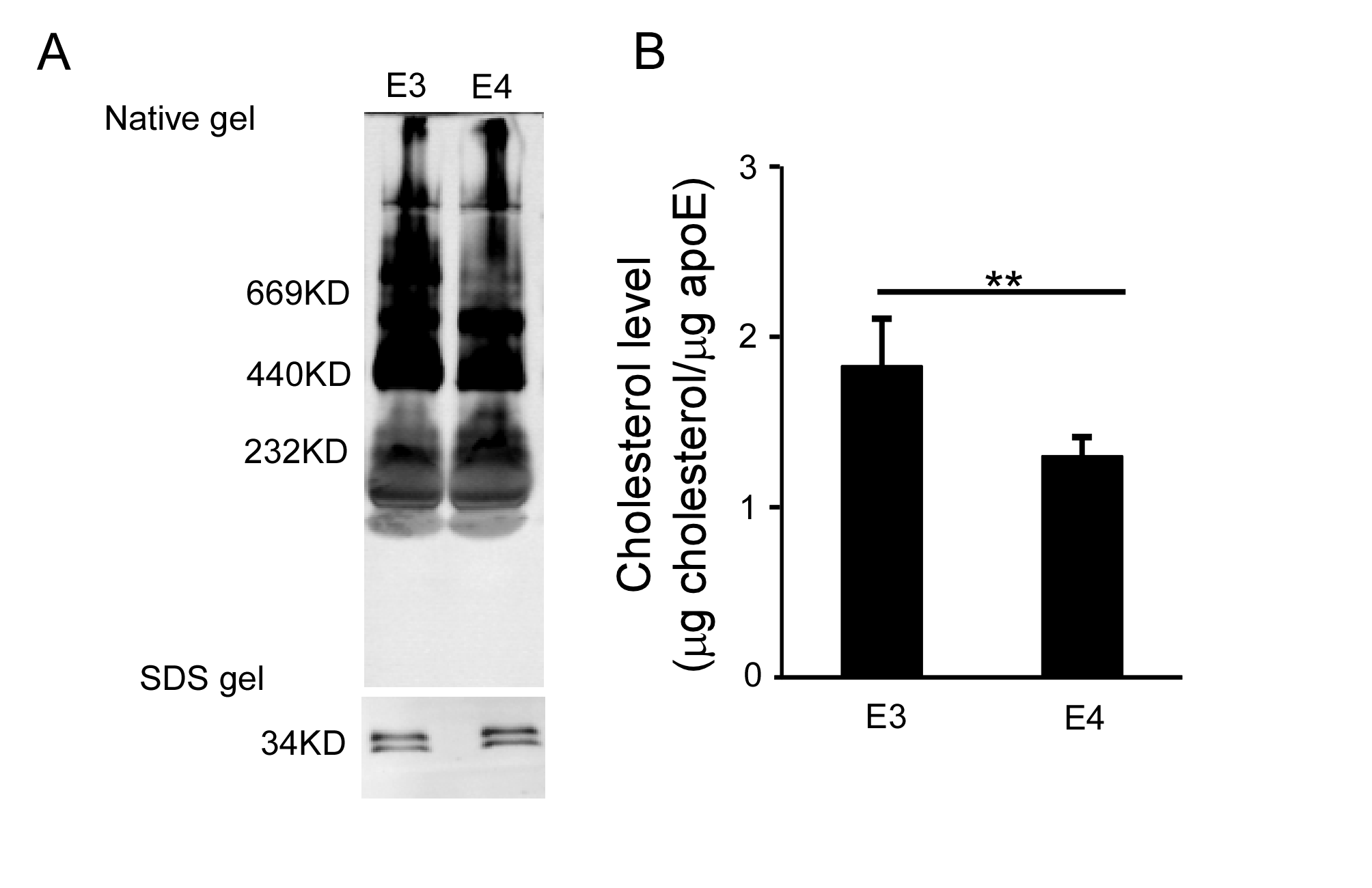

Supplement: Additional file 1: Figure S1. — ApoE particles secreted by primary astrocytes from apoE-TR mice. (A) ApoE3 and apoE4 particles were isolated from condition medium of primary astrocytes from apoE3-TR and apoE4-TR mice using immunoaffinity column. The purified apoE3 (500 ng) and apoE4 particles (500 ng) were analyzed by non-denaturing gradient gel electrophoresis (4–20 %) and SDS-PAGE, followed by Western blot for apoE. (B) Total cholesterol concentrations of the apoE3 and apoE4 particles were measured using the Amplex Red cholesterol assay kit and normalized against apoE concentrations. Data represent mean ± S.D. (n = 3). **, p < 0.001. (TIF 213 kb) [file 13024_2016_99_MOESM1_ESM.tif]

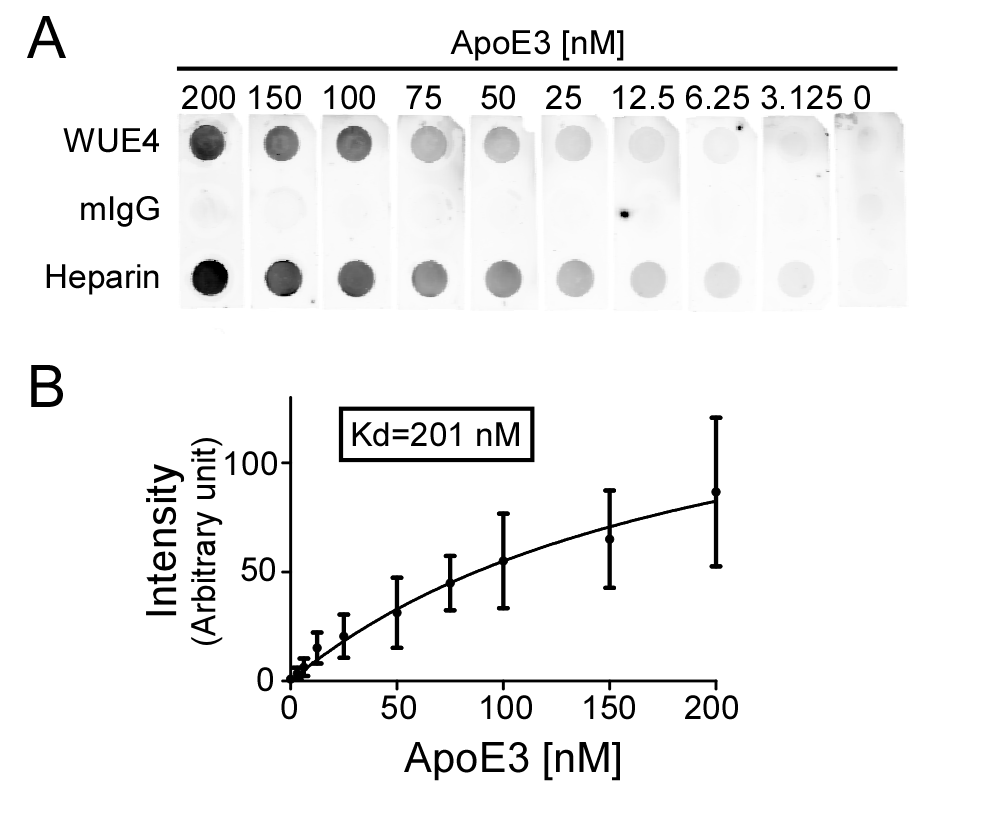

Supplement: Additional file 2: Figure S2. — Binding affinity of heparin-apoE3 interaction. (A) Representative dot blot of heparin and apoE3 particles. Heparin was spotted onto nitrocellulose membrane along with mouse monoclonal anti-apoE antibody, WUE4, as a positive control and normal mouse IgG as a background. Membrane strips were incubated with increasing concentrations of apoE3 particles from immortalized astrocytes. Membrane-bound apoE was then visualized by biotin-conjugate anti-apoE antibody and infrared streptavidin secondary antibody. (B) Integrated infrared signal intensities from each dot were obtained and the average intensities from three independent experiments were plotted to acquire binding affinity curve and the dissociation constant (Kd). (TIF 2432 kb) [file 13024_2016_99_MOESM2_ESM.tif]

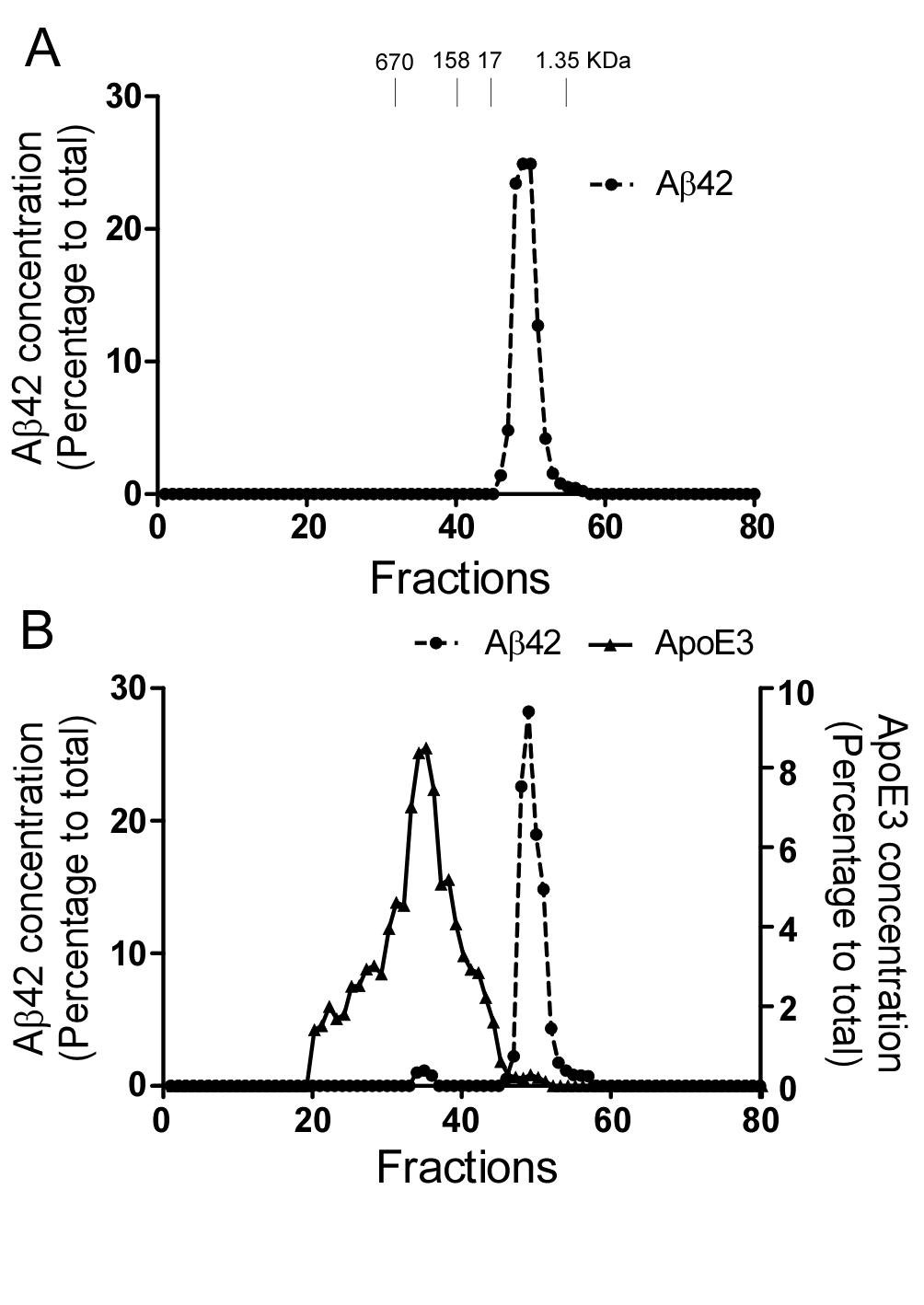

Supplement: Additional file 3: Figure S3. — ApoE particles are minimally associated with Aβ. Aβ was incubated without (A) or with apoE3 particles (B). Samples were applied to SEC with tandem Superose-6 columns. Concentrations of apoE and Aβ in each fraction were analyzed by ELISA. Graphs are averaged from separate experiments (n = 3). (TIF 4171 kb) [file 13024_2016_99_MOESM3_ESM.tif]

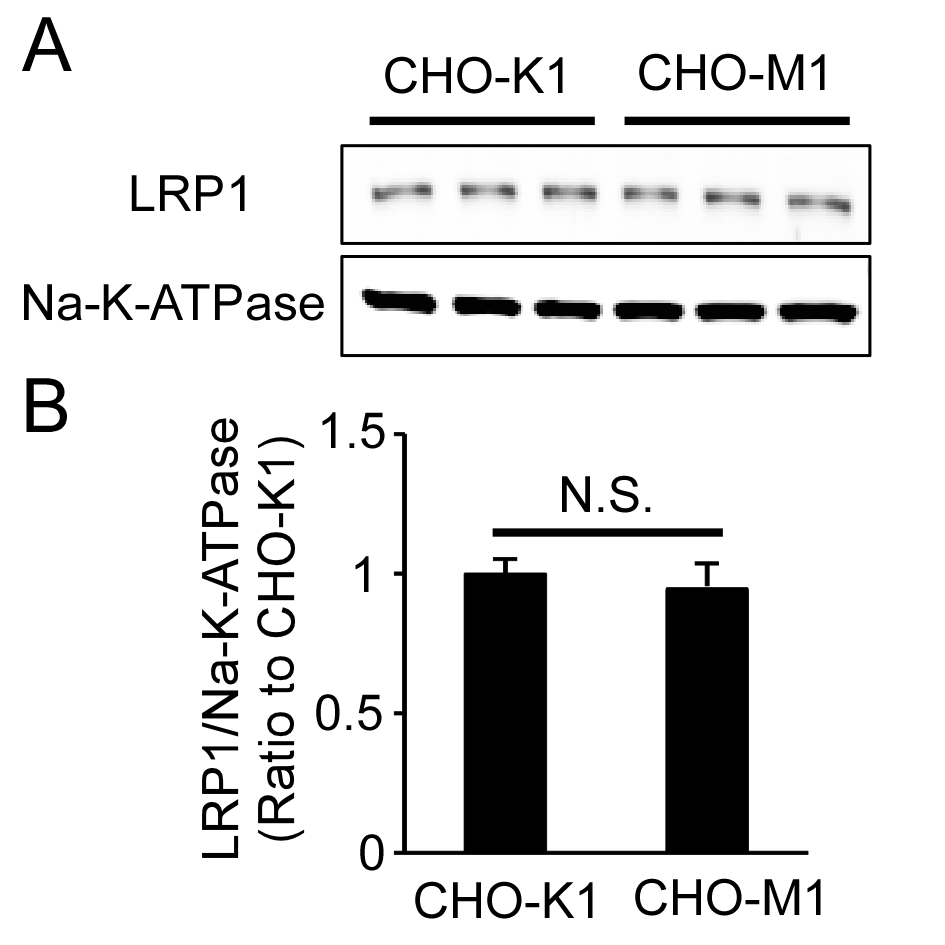

Supplement: Additional file 4: Figure S4. — Similar LRP1 levels in the membrane fractions of CHO-K1 cells and CHO-M1 cells. Membrane fractions isolated from CHO-K1 and CHO-M1 cells were subjected to Western blot for LRP1 and Na-K-ATPase (A). The levels of LRP1 were normalized against those of Na+-K+-ATPase and plotted in (B). Data represent mean ± S.D. (n = 3). N.S., not significant. (TIF 63 kb) [file 13024_2016_99_MOESM4_ESM.tif]
